# Supplementary material for: Structural puzzles in virology solved with an overarching icosahedral design principle
Source: Nat Commun. 2019 Sep 27;10:4414. doi: 10.1038/s41467-019-12367-3 (PMC6765026; doi:10.1038/s41467-019-12367-3)
Supplement: Supplementary file 1 — Supplementary Information [file 41467_2019_12367_MOESM1_ESM.pdf]

Structural puzzles in virology solved with an overarching icosahedral design principle

Reidun Twarock[1,⋆] and Antoni Luque[2,⋆]

[1] Departments of Mathematics and Biology, York Cross-disciplinary Centre for Systems Analysis, University of York, York YO10 5GE, UK

[2] Department of Mathematics and Statistics, Viral Information Institute, and Computational Science Research Center, San Diego State University, 5500 Campanile Drive, San Diego, CA 92182-7720, US

⋆ joint corresponding authors,

emails: reidun.twarock@york.ac.uk and aluque@sdsu.edu

Supplementary Material

Supplementary Figures

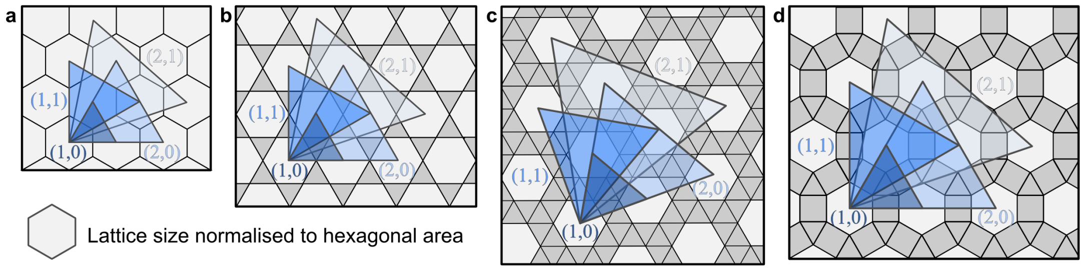

Supplementary Figure 1: The Caspar Klug construction for the Archimedean lattices. The triangular icosahedral units corresponding to the hexagonal coordinates h and k used in the construction of the polyhedral shapes in the different lattices: **a** hexagonal (as in CK theory) $(6, 6, 6)$; **b** trihexagonal $(3, 6, 3, 6)$; **c** snub hexagonal $(3^4, 6)$; and **d** rhombitrihexagonal $(3, 4, 6, 4)$. In particular, a triangle connecting the midpoints of three adjacent hexagons $(h, k) = (1, 0)$ in the (sub)lattice contains: **a** none; **b** one triangle; **c** four triangles; and **d** one triangle and $\frac{3}{2}$ squares.

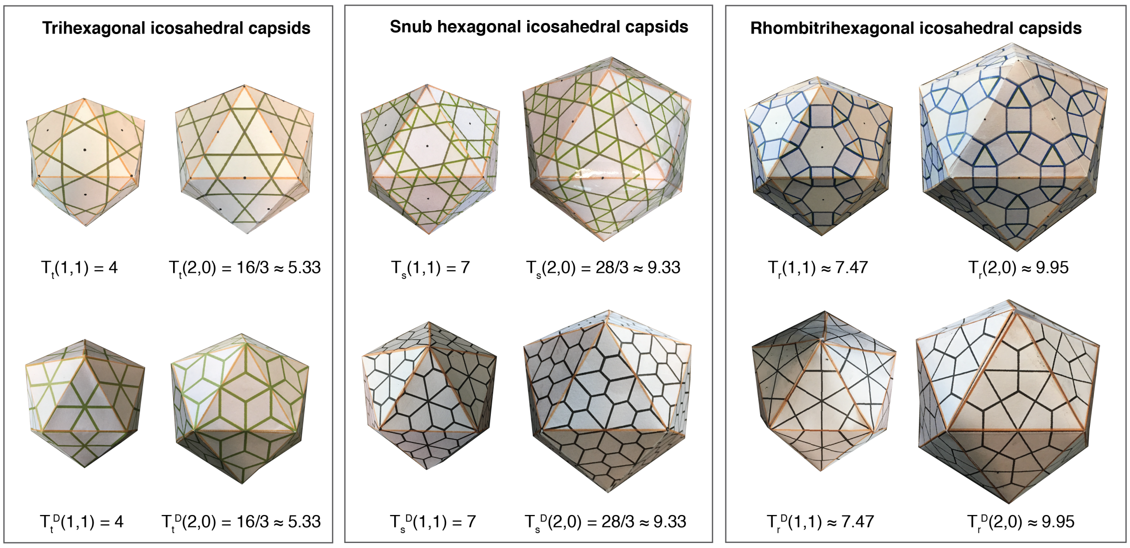

Supplementary Figure 2: The start of the icosahedral series and their duals. Models of the icosahedral surface lattices constructed as in (1) for the trihexagonal, snub hexagonal and rhombihexagonal icosahedral capsid series (top row) and their duals (bottom row) are shown, together with the corresponding labels according to (2).

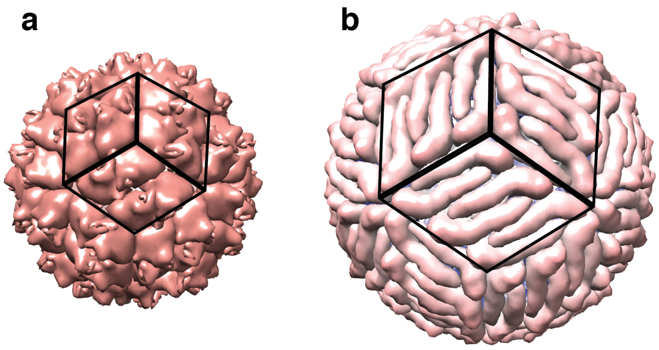

Supplementary Figure 3: Non-quasiequivalent capsid achitectures with higher-order rhomb-like tiles. The icosahedral design principle covers also quasiequivalent arrangements of identical copies of non-quasiequivalent environments that represent multiple proteins of a similar type. **a** Picobirnavirus displays a rhomb tiling ($T_t^D(1,0)$) with four proteins per rhomb tile (2 parallel dimers) (PDB 2VF1)[1]. **b** Another example is the rhomb tiling representing Zika virus ($T_t^D(1,0)$ with six proteins per rhomb tile (3 parallel dimers) (PDB 5IZ7) [2].

Supplementary Tables

The following tables list the numbers of pentagonal, hexagonal, triangular and square faces of each icosahedral polyhedron. They also include the numbers of proteins in the corresponding viral architecture model. Supplementary Table 1 corresponds to the geometries predicted in Caspar and Klug's quasiequivalence theory. Supplementary Tables 2–5 correspond to the additional geometries introduced by the new geometrical framework presented here.

(h,k)	T	n_{hex}	n_{pent}	n_p
(1,0)	1	0	12	60
(1,1)	3	20	12	180
(2,0)	4	30	12	240
(2,1)	7	60	12	420
(3,0)	9	80	12	540
(2,2)	12	110	12	720
(3,1)	13	120	12	780

Supplementary Table 1: The icosahedral hexagonal lattice architectures. This table lists the first elements of the polyhedral series in CK theory. There are $n_p = 60T$ proteins organised into $n_{hex} = 10(T-1)$ hexamers and $n_{pent} = 12$ pentamers. Capsomers are oriented edge-to-edge, that is, pairs of proteins in a hexamer or pentamer meet pairs of proteins in another hexamer or pentamer (Supplementary Figure 1**a**). In the case of an octahedral particle constructed from an octahedral surface superimposed on a hexagonal lattice, there are $n_p = 24T$ putative protein positions, with $n_{hex} = 4(T-1)$ organised in hexamers, and another 24 proteins in clusters of four at the six 4-fold axes. The latter, however, are likely unoccupied due to stress created by strong curvature.

(h,k)	T_t	n_{hex}	n_{tri}	n_{pent}	n_p^{MCP}	n_p^{mCP}
(1,0)	4/3	0	20	12	60	60
(1,1)	4	20	60	12	180	180
(2,0)	16/3	30	80	12	240	240
(2,1)	28/3	60	140	12	420	420
(3,0)	12	80	180	12	540	540
(2,2)	16	110	240	12	720	720
(3,1)	52/3	120	260	12	780	780

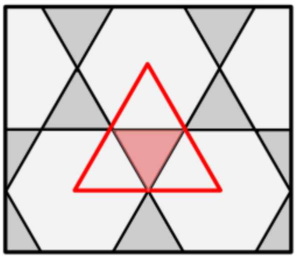

Supplementary Table 2: The trihexagonal lattice architectures. The trihexagonal number, $T_t(h,k)$, is proportional to the classic triangulation number, $T(h,k)$, as given in Eq. (2). The associated icosahedral structures contain $n_{hex} = 10(T-1)$ hexagonal, $n_{pent} = 12$ pentagonal, and $n_{tri} = 20T$ triangular faces. There are at least two distinct ways in which this layout can

be realized by a viral geometry: $n_p^{MCP} = 60T$ counts proteins for the case that the triangular positions are occupied by a domain of a major capsid protein (MCP); in this case, the number of proteins is as for the Caspar-Klug geometries, but their orientations are different (see below). $n_p^{mCP} = 60T$ counts the additional proteins for the case that the triangular positions are occupied by an additional minor capsid protein (mCP). Capsomer orientations are vertex-to-vertex, that is individual proteins in a hexamers or pentamer meet individual proteins in another hexamer or pentamer (Supplementary Figure 1**b**). In the case of an octahedral particle constructed from an octahedral surface superimposed on a trihexagonal lattice, there are $n_p = 24T$ putative protein positions, with $n_{hex} = 4(T-1)$ organised in hexamers, $n_{tri} = 8T$ organised in trimers, and another 24 proteins in clusters of four at the six 4-fold axes. The latter, however, are likely unoccupied due to stress created by strong curvature.

(h,k)	T_s	$\mathrm{n_{hex}}$	$\mathrm{n_{tri}}$	$\mathrm{n_{pent}}$	$\mathrm{n_p^{MCP}}$	$\mathrm{n_p^{mCP}}$
(1,0)	7/3	0	80	12	120	60
(1,1)	7	20	240	12	360	180
(2,0)	28/3	30	320	12	480	240
(2,1)	49/3	60	560	12	840	420
(3,0)	21	80	720	12	1080	540
(2,2)	28	110	960	12	1440	720
(3,1)	91/3	120	1040	12	1560	780

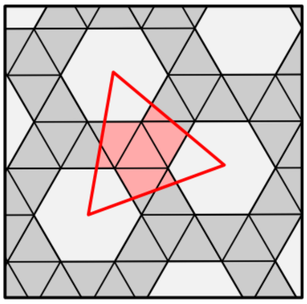

Supplementary Table 3: The snub hexagonal lattice architectures. The snub hexagonal number, $T_s(h,k)$, is proportional to the classic triangulation number, $T(h,k)$, as given in Eq. (2). The associated icosahedral structures contain $n_{hex} = 10(T-1)$ hexagonal, $n_{pent} = 12$ pentagonal, and $n_{tri} = 80T$ triangular faces. There are several distinct ways in which this layout can be realized by a viral geometry: pentamers/hexamers of dimers (covering pentagons/hexagons and adjacent triangles) as MCP, and the remaining triangular position either empty ($n_{MCP} = 6n_{hex} + 5n_{pent} + 60T$), where the remaining triangular positions can be occupied by CP domains in the nearby triangles. Alternatively, these positions could be occupied by 3 mCPs each, that is, $n_{mCP} = 60T$ mCPs in the capsid. MCP can also group in a different way, with 60 proteins in pentamers, $60(T-1)$ in hexamers, $30T$ in dimers (that are composed of two triangles, each representing a monomer), with the remaining $20T$ triangles either unoccupied, or occupied by $60T$ mCP. Capsomer orientations are edge-to-edge, that is pairs of proteins in a hexamers or pentamer meet pairs of proteins in another hexamer or pentamer, but with a parallel shift (shear) (Supplementary Figure 1**c**). In the case of an octahedral particle constructed from an octahedral surface superimposed on a snub

hexagonal lattice, there are $n_{hex} = 4(T-1)$ proteins organised in hexamers and $n_{tri} = 32T$ organised in trimers, and another 24 proteins in clusters of four at the six 4-fold axes. The latter, however, are likely unoccupied due to stress created by strong curvature.

(h,k)	T_r	$\mathrm{n_{hex}}$	$\mathrm{n_{tri}}$	$\mathrm{n_{pent}}$	$\mathrm{n_{square}}$	$\mathrm{n_p^{MCP}}$	$\mathrm{n_p^{mCP}} = \mathrm{n_p^{mCP2}}$
(1,0)	$(4/3 + \frac{2}{\sqrt{3}})$	0	20	12	30	60	60
(1,1)	$(4 + 2\sqrt{3})$	20	60	12	90	180	180
(2,0)	$(16/3 + \frac{8}{\sqrt{3}})$	30	80	12	120	240	240
(2,1)	$(28/3 + \frac{14}{\sqrt{3}})$	60	140	12	210	420	420
(3,0)	$(12 + 6\sqrt{3})$	80	180	12	270	540	540
(2,2)	$(16 + 8\sqrt{3})$	110	240	12	360	720	720
(3,1)	$(52/3 + \frac{26}{\sqrt{3}})$	120	260	12	390	780	780

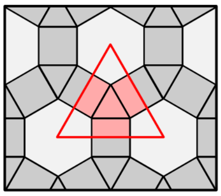

Supplementary Table 4: The rhombitrihexagonal lattice architectures. The rhombitrihexagonal number, $T_r(h,k)$, is proportional to the classic triangulation number, $T(h,k)$, as given in Eq. (2). The associated icosahedral structures contain $n_{hex} = 10(T-1)$ hexagonal, $n_{pent} = 12$ pentagonal, $n_{tri} = 20T$ triangular, and $n_{square} = 30T$ square faces. There are three distinct ways in which these layouts can be realized by a viral geometry: $n_p^{MCP} = 60T$ counts proteins for the case that the triangular and square positions are occupied by a domain of a major capsid protein (MCP) each; in this case, the number of proteins is as for the Caspar-Klug geometries, but in addition, there are two distinguished domains playing special roles. If instead the triangular positions are occupied by 3 mCPs or (3 reinforcement proteins) each, then there are in addition $n_p^{mCP} = 60T$ mCPS or reinforcement proteins. Finally, if instead the square positions are occupied by dimers of a (potentially different) mCP, then there are moreover $n_p^{mCP2} = 60T$ mCP2s. Capsomer orientations are edge-to-edge, that is pairs of proteins in a hexamers or pentamer meet pairs of proteins in another hexamer or pentamer (Supplementary Figure 1**d**). In the case of an octahedral particle constructed from an octahedral surface superimposed on a rhombitrihexagonal lattice, there are $n_{hex} = 4(T-1)$ proteins organised in hexamers, and $n_{tri} = 8T$ organised in trimers. There are another $n_{square} = 12T$ proteins organised in quatromers, and 24 proteins in clusters of four at the six 4-fold axes. However, the latter, and potentially also the quatromers, are likely unoccupied due to stress created by strong curvature.

(h,k)	n_t^r	n_s^f	n_r^k
(1,0)	30	60	60
(1,1)	90	180	180
(2,0)	120	240	240
(2,1)	210	420	420
(3,0)	270	540	540
(2,2)	360	720	720
(3,1)	390	780	780

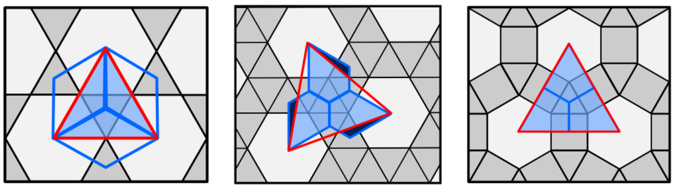

Supplementary Table 5: The duals of the trihexagonal, snub hexagonal, and rhombitrihexagonal lattice architectures. Using h and k as the coordinates in the hexagonal sublattice of the trihexagonal, snub hexagonal and hexadille lattice as in Eq. (2), and $T = h^2 + hk + k^2$, there are $n_t^r = 30T$ rhombs in the dual trihexagonal polyhedra, $n_s^f = 60T$ floret tiles in the dual snub hexagonal polyhedra, and $n_r^k = 60T$ kite tiles in the dual rhombitrihexagonal polyhedra. If rhombs are occupied by 2 proteins, and kites and florets by a singe protein, then there are $60T$ proteins in each case. It is also possible that each tile is occupied by several copies of the same protein unit, if the shapes of these units are consistent with the shape of the tile. For example, in the case of the rhomb tile these are units arranged parallel to one of its edges that collectively fill the surface area of the tile. In the case of the floret tiles, these are six identical proteins, five arranged as a pentamer and one further protein at the pointed angle, that either contributes to a pentamer or a hexamer in the tiling depending on its position, resulting in $12 + 60T$ pentamers and $10(T-1)$ hexamers. For the kite tile, there are three proteins, two at the top and one corresponding to the pointed angle, thus corresponding to 12 pentamers and $10(T-1) + 20T = 30T - 10 = 10(3T-1)$ hexamers. In the case of an octahedral particle constructed from an octahedral surface superimposed on a rhombitrihexagonal lattice, there are $n_t^r = 12T$ rhombs in the dual trihexagonal polyhedra, $n_s^f = 24T$ floret tiles in the dual snub hexagonal polyhedra, and $n_r^k = 24T$ kite tiles in the dual rhombitrihexagonal polyhedra.

Supplementary Discussion

Viral capsids belonging to the same structural lineage, i.e. with similar capsid protein folds, can display different lattice types if their capsid protein domains adopt similar, yet distinct, conformations. For fixed numbers of capsid proteins, the relative radii of the capsids are implied by their lattice geometries and can therefore be indicators of lattice types. An example are Tobacco ringspot virus (a rhombitrihexagonal dual lattice architecture, $T_r^D(1,0) = \alpha_r T(1,1) = 3\alpha_r$) and Pariacoto virus (a hexagonal dual lattice architecture, $T^D(1,1) = T(1,1) = 3$), that both belong to the single

jelly roll lineage. Their surfaces can be expressed as $S_T = 60T_r^D(1,0)s_0$ and $S_P = 60T^D(1,1)s_0$, where the indices T and P identify Tobacco ringspot virus and Pariacoto, respectively, and s_0 is a surface factor that is assumed to be the same for both, because both viruses belong to the same lineage. Expressing both relations in terms of the regular T-number to facilitate comparison, one has $S_T = 60\alpha_r T(1,0)s_0$ and $S_P = 60T(1,1)s_0$. The surface ratio can then be related to their radii by $S_T/S_P = (R_T/R_P)^2$, which leads to the capsid size ratio

$$\frac{R_T}{R_P} = \sqrt{\frac{\alpha_j}{3}} \ . \tag{1}$$

If both capsids followed the same lattice architecture, the ratio between their radii would be $\sqrt{1/3}$. However, as explained in the main text, experimental data reveal $\alpha_j > 1$, consistent with Tobacco ringspot virus conforming to a rhombitrihexagonal dual lattice architecture, rather than the hexagonal dual lattice architecture of Pariacoto virus.

References

[1] Stéphane Duquerroy, Bruno Da Costa, Céline Henry, Armelle Vigouroux, Sonia Libersou, Jean Lepault, Jorge Navaza, Bernard Delmas, and Félix A Rey. The picobirnavirus crystal structure provides functional insights into virion assembly and cell entry. *The EMBO Journal*, 28:1655–1665, (2009).

[2] V.A. Kostyuchenko, E.X.Y. Lim, S. Zhang, G. Fibriansah, T.-S. Ng, J.S.G. Ooi, J. Shi, and S.-M. Lok. Structure of the thermally stable zika virus. *Nature*, 533:425–428, (2016).
